# Pain intensity and cervical range of motion in women with myofascial pain treated with acupuncture and electroacupuncture: a double-blinded, randomized clinical trial

**DOI:** 10.1590/bjpt-rbf.2014.0066

**Published:** 2015

**Authors:** Maria F. M. Aranha, Cristina E. E. Müller, Maria B. D. Gavião

**Affiliations:** 1 Departamento de Morfologia, Faculdade de Odontologia de Piracicaba (FOP), Universidade Estadual de Campinas (UNICAMP), Piracicaba, SP, Brazil; 2 Departamento de Odontologia Infantil, FOP, UNICAMP, Piracicaba, SP, Brazil

**Keywords:** myofascial pain syndromes, acupuncture therapy, trapezius muscle, physical therapy

## Abstract

**BACKGROUND:**

Acupuncture stimulates points on the body, influencing the perception of myofascial pain or altering physiologic functions.

**OBJECTIVE:**

The aim was to evaluate the effect of electroacupuncture (EAC) and acupuncture (AC) for myofascial pain of the upper trapezius and cervical range of motion, using SHAM acupuncture as control.

**METHOD:**

Sixty women presenting at least one trigger point at the upper trapezius and local or referred pain for more than six months were randomized into EAC, AC, and SHAM groups. Eight sessions were scheduled and a follow-up was conducted after 28 days. The Visual Analog Scale assessed the intensity of local and general pain. A fleximeter assessed cervical movements. Data were analyzed using paired t or Wilcoxon's tests, ANOVA or Friedman or Kruskal-Wallis tests and Pearson's correlation (α=0.05).

**RESULTS:**

There was reduction in general pain in the EAC and AC groups after eight sessions (*P*<0.001). A significant decrease in pain intensity occurred for the right trapezius in all groups and for the left trapezius in the EAC and AC groups. Intergroup comparisons showed improvement in general pain in the EAC and AC groups and in local pain intensity in the EAC group (*P*<0.05), which showed an increase in left rotation (*P=*0.049). The AC group showed increases in inclination (*P=*0.005) sustained until follow-up and rotation to the right (*P=*0.032).

**CONCLUSION:**

: EAC and AC were effective in reducing the pain intensity compared with SHAM. EAC was better than AC for local pain relief. These treatments can assist in increasing cervical range of motion, albeit subtly.

## Introduction

Myofascial pain is characterized by the presence of tender, firm nodules called trigger points (MTrP). Within each trigger point is a hyperirritable spot, the "taut-band", which is composed of hyper-contracted muscle fibers^1^. Clinically, the muscle with MTrP presents with stiffness and is associated with diminished strength and restricted range of motion^1^^,^ 2. If latent, palpation of this spot within the trigger point provokes radiating, aching-type pain into localized referred zones consisting in an important musculoskeletal dysfunction^3^ and one of main causes of headache and neck pain^4^. If active, MTrP promotes spontaneous pain.

Myofascial pain affects up to 85% of the general population^1^. Fleckenstein et al.^5^ reported that physicians did not observe difference between genders. Nevertheless, women were more likely than men to develop neck pain and less likely to recover from such pain^6^. Moreover, women have a greater frequency of musculoskeletal pain than men^7^.

The treatment of myofascial pain requires that MTrP and muscles be identified as primary or ancillary pain generators^3^. Acupuncture (AC) has been used as an alternative to more traditional treatments for musculoskeletal pain, because it inactivates the neural loop of the trigger point (pain-contraction-pain), reducing pain, and reduces muscular over-contraction. Acupuncture stimulates points on the body via the insertion of needles to prevent or modify the perception of pain or to alter physiologic functions^8^. Electroacupuncture (EAC) includes the passage of an electrical current through the needle^9^.

AC^8^^,^ 10 and EAC^9^^,^ 11 ^,^ 12 have been shown to effectively decrease the intensity of chronic pain. Nevertheless, it is still unknown whether one of these treatments is more effective than the other for treating myofascial pain. Since EAC presents the electrical stimulation added to AC, better results are expected.

Myofascial pain treatment should increase muscle function^2^, promoting pain decrease and the unstringing of the contracted fibers. Consequently, a reduction in muscle stiffness and an increased range of motion would be expected.

Fiber contraction in the upper trapezius muscle, which presents a high prevalence of MTrP^13^^-^ 15, promotes the extension and inclination of the head to the same side and rotation to the opposite side^16^. Therefore, to evaluate muscle function, cervical range of motion is indicated. To achieve that, non-invasive methods are available such as the *Cervical Range of Motion* device (Roseville, MN, USA)^17^ and the fleximeter (Code Research Institute, Brazil)^18^, which have presented moderate to excellent reliability for both intra- and inter-examiner measurements. Although both methods are effective, the fleximeter offers lower costs and easier handling, and it can evaluate other body segments.

The aim of this study was to evaluate the effectiveness of EAC and AC on pain intensity and cervical range of motion in women with myofascial pain in the upper trapezius, using SHAM acupuncture as a control. The hypothesis was that EAC could determine better effectiveness than AC in pain relief and increase the cervical range of motion.

## Method

This research was conducted at the Clinical Research Laboratory, Departamento de Odontologia Infantil, Faculdade de Odontologia de Piracicaba, Universidade Estadual de Campinas (FOP-UNICAMP), Piracicaba, SP, Brazil, from June 2012 to August 2013. The sample size was calculated considering previous data for pain intensity^12^. A two-sided test with α=0.05, null mean=0.5, alternate mean=0, standard deviation=2.5, and statistical power=0.80, determined a sample size of 24 subjects for each group. The project was approved by the Research Ethics Committee of FOP-UNICAMP (protocol 003/2011). The volunteers read and signed the consent form and were informed about the procedures, discomfort or risks, the benefits of the research, and the need to attend all sessions. They were blinded to group allocation, as was the examiner who did the assessments (C.E.E.M.). The Brazilian Clinical Trials Registry number is RBR-42kz9z (available at: http://www.ensaiosclinicos.gov.br/rg/RBR-42kz9z/).

Women suffering from head and neck pain were included in the study. The participants were interviewed to obtain information about general health pertaining to the inclusion and exclusion criteria, as follows: (1) Inclusion criteria: age range from 18 to 40 years; body mass index (BMI) ranging from 18 to 30 Kg/m^2^; regular menstrual cycle (regardless of oral contraception use); and at least one active MTrP in the upper trapezius muscle, with spontaneous local or referred persistent pain for at least six months. (2) Exclusion criteria: accentuated postural abnormalities (verified by physical therapist C.E.E.M.), fibromyalgia syndrome, cervical radiculopathy, systemic disease or physical therapy interventions for myofascial pain within one month before the study, pregnancy, chronic pacemaker or electronic implant use (reported by the subject). Latent MTrP was not an exclusion criterion. The continuous use of medications for headache and muscular pain was also an exclusion criterion. Moreover, subjects with evident cognitive impairment or communication difficulties during the first meeting were excluded.

**Figure 1:**
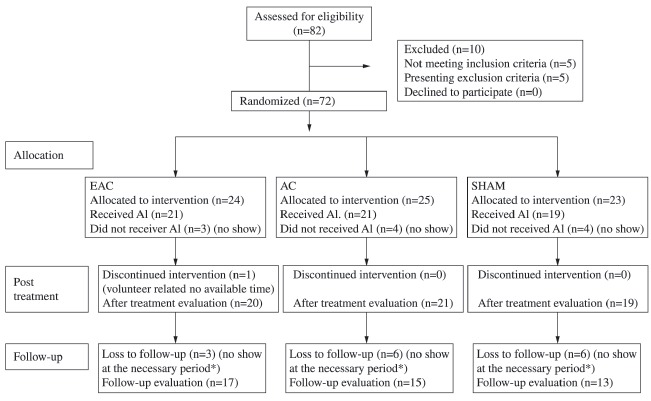
Enrollment of participants and study design. Al = Allocated intervention; *refers to menstrual cycle period.

Initially, 82 volunteers were eligible. Ten were not included: two above BMI limit; one without active MTrPs; one with pain for less than six months, one without a regular menstrual cycle; one pregnant, one with a cervical hernia; one with trigeminal neuralgia; two with fibromyalgia. Seventy-two volunteers were included. Of these, seven dropped out before the first session and five started but did not complete the eight treatment sessions (Figure 1). Consequently, the statistical power was reconsidered as 0.70, according to the parameters cited above, determining a minimum of 20 subjects for each group. Nevertheless, in the SHAM group, 19 volunteers completed the treatment.

Women should be evaluated in the same phase of their cycle because both absolute and relative hormone levels could influence pain^19^. Thus, measurements (pre-, post-treatment, and follow-up) were fixed between the second and the fifth day of menstruation period, with 28 days between each measurement. Between evaluation and reevaluation, eight sessions were scheduled, two per week. Reevaluation was scheduled three to six days after the last session, coinciding with the second to the fifth day of the volunteer's menstrual phase.

All volunteers were diagnosed as having active MTrPs bilaterally. Each side was analyzed separately. They were distributed among three groups: EAC, AC, and SHAM. First, the volunteers were coded by the blinded examiner according to their use of oral contraceptives: paused, continuous or without oral contraception. After that, they were randomly allocated to each group using Excel. Therefore, all volunteers had the same chance of being allocated to any group, avoiding the predominance of one oral contraceptive condition among groups. Right was the dominant side. One volunteer was left-handed (EAC group).

### Instrumentation

The device used for the EAC was the EL608 NKL (ANVISA 80191680002). The needles were stainless steel, individually wrapped, sterile, and disposable, with a diameter of 0.25 mm and a length of 30 mm (Dong-Bang, Korea).

The Visual Analog Scale (VAS) assessed the intensity of general pain and pain in the right and left upper trapezius. The scale consists of an unanchored horizontal line 10 centimeters in length, with one end corresponding to zero ("no pain") and the other to 10 ("maximum pain").

The fleximeter, used for cervical motion measurements, consisted of a gravity-dependent inclinometer with a graduated scale attached to the head with Velcro tap (Code Research Institute, Brazil).

To monitor intercurrences between sessions, an additional data form, consisting of open-ended questions about trauma, headaches, neck and shoulder pain, medications (such as analgesics, non-steroidal anti-inflammatories, and spasmolytic drugs) and the respective doses, was applied at the beginning of the sessions. Additionally, emotional stress conditions that could occur between sessions were considered because muscle tension can be an expression of anxiety and emotional tension^1^. Such conditions were considered influencing factors, i.e. they could interfere with the treatment effects.

### Procedures

The diagnosis of MTrP was based on five criteria^1^^,^ 3: (1) the presence of a palpable taut band in the muscle; (2) the presence of a hypersensitive tender spot in a taut band; (3) a local twitch response elicited by the snapping palpation of the taut band; (4) reproduction of the typical referred pain pattern of the MTrP in response to compression; (5) the spontaneous presence of the typical referred pain pattern and/or recognition of the referred pain as familiar. MTrP was considered active if the referred pain, whether spontaneous or evoked by compression, reproduced the subject's complaint; MTrP was considered latent if the referred pain did not reproduce a usual or familiar pain. The volunteer remained seated on a chair during the examination.

Both VAS and fleximetry were measured pre- and post-treatment and at the follow up. Volunteers were asked to mark their pain between "no pain" and "maximum pain" on the printed VAS. Afterwards, the marked location was measured with a ruler (in centimeters) by the blinded examiner.

The head and neck movements evaluated were: flexion, extension, inclination to the right and to the left, and rotation to the right and to the left. All movements except rotation were measured with the subjects seated on a chair with the back straight, eyes looking straight ahead and parallel to the floor, knees flexed at 90 degrees and feet flat on the floor. For the rotation movements, the volunteers had to stay in the supine position to keep the fleximeter favorably positioned relative to the effects of gravity.

During EAC application, the patient remained in the prone position. Needles were inserted bilaterally into points GB21 and GB20 (local analgesic acupoints) and unilaterally into LI4, LV3^20^ (distal analgesic acupoints), and a maximum of two needles on each side directly in the region of the "Ashi Points" (painful points not predicted on meridians, not necessarily MTrP, detected before each session according to subject report at soft palpation of muscle). The equipment was programmed as follows: alternating frequency F1=2 Hz, T1=5 seconds, F2=100 Hz, T2=5 seconds; total time: 30 minutes; intensity: maximum supported by the patient without pain^12^^,^ 21. The acupuncture group received the same treatment but without the connection to the alternating frequency equipment. The SHAM acupuncture group had the needles inserted 1 cm distally from the correct acupoints.

### Statistics

The assumptions of equality of variances and normal distribution were checked for all variables (Shapiro-Wilk test). Intragroup comparisons were analyzed using Student's paired t-test or Wilcoxon's signed rank test, one-way repeated measures ANOVA or the Friedman repeated measures with the Student-Newman-Keuls method for *post-hoc* analysis. To identify intergroup differences, one-way ANOVA or the Kruskal-Wallis test was used, with Dunn's Method as the *post-hoc* analysis. Moreover, analysis of variance was applied based on a generalized linear mixed model for two factors: group (fixed) and time of evaluation as repeated measures. This analysis used the t-test adjusted with the Tukey-Kramer test. Pearson's correlation was applied and SigmaPlot (Systat Software, San Jose, CA, USA) was used. A generalized linear mixed model was developed with SAS System (SAS Institute Inc, release 9.3. SAS Institute Inc., Cary, NC, USA; 2010).

## Results

The final sample consisted of 60 females. Their mean age was 27.33±4.95 years, and their BMI ranged from 19.31 to 25.79 Kg/m^2^ (22.55±3.24 Kg/m^2^). All volunteers were diagnosed with active MTrPs bilaterally. Each side was analyzed separately. The sample distribution in accordance to oral contraceptive use was similar among treatment groups. For paused and continuous oral contraception, eight and four volunteers were allocated to the EAC, AC, SHAM groups, respectively. The corresponding values for those without oral contraception were eight, nine, and seven.

Forty-five females attended the follow-up session. Their mean age and BMI were 26.73±4.76 years and 22.58±3.30 Kg/m^2^, respectively.

### Pain intensity

### Pre-treatment (Figure 2A, B, C)

**Figure 2:**
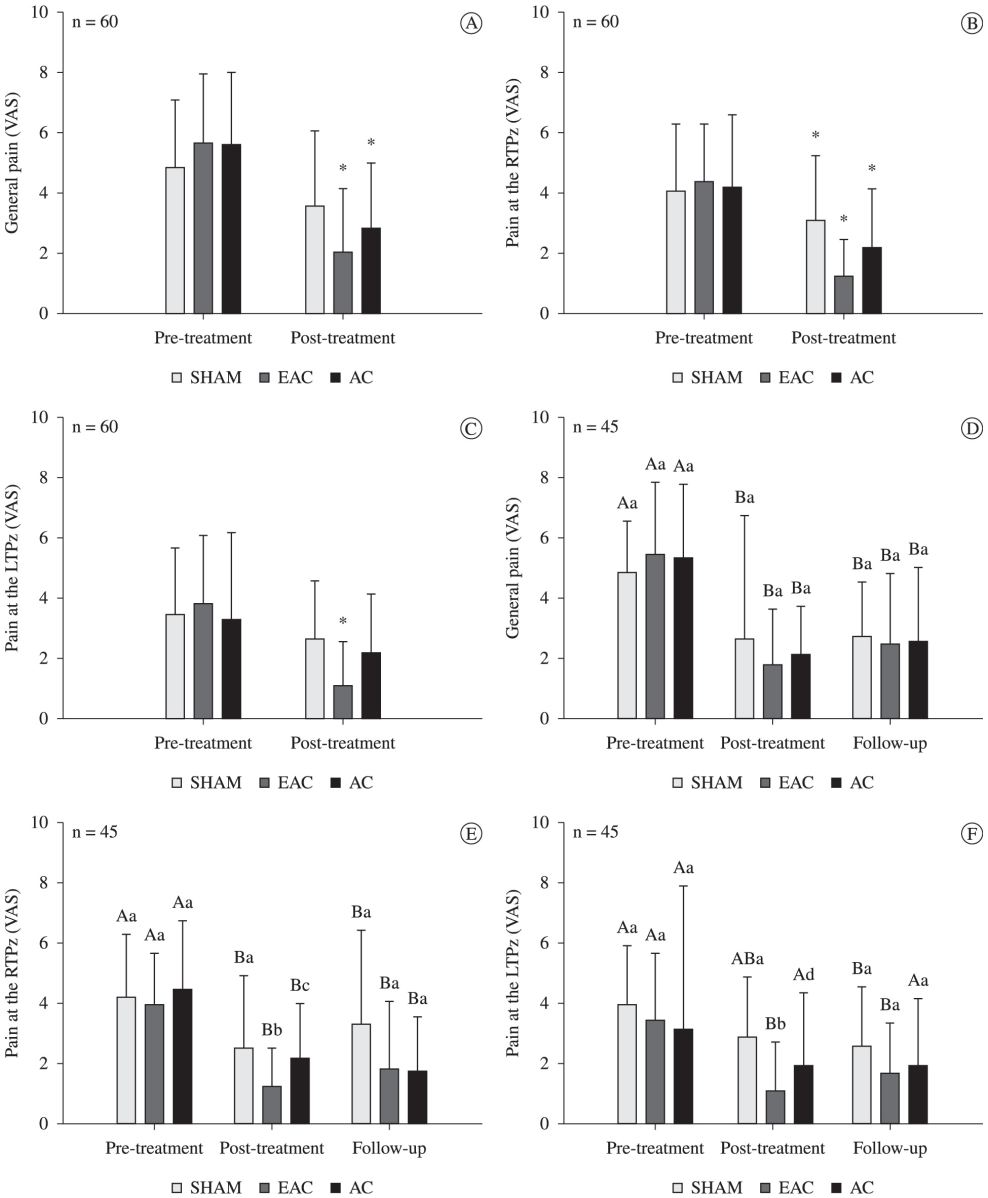
Means and standard deviations for general pain (A), pain at the right upper trapezius (B) and at the left upper trapezius (C) in pre- and post-treatment and including the follow-up evaluation (D, E, F). Uppercase letters above the standard deviation bars refer to intragroup comparisons: A?B (P<0.05); lowercase letters refer to intergroup comparisons: c: AC=EAC and AC=SHAM; d: SHAM=AC and AC?EAC; a?b*(P<0.05).

The comparisons among the three treatment groups in the same moment showed that in the pre-treatment evaluation, there were no differences among groups concerning general pain (*P=*0.493) or pain in the right upper trapezius (*P=*0.908) or the left upper trapezius (*P=*0.723), indicating the homogeneity of the groups.

The basal pain values showed no differences according to oral contraceptive conditions.

### Post-treatment (Figure 2A, B, C)

As Figure 2 presents, only the EAC and AC groups showed significant decreases in general pain after treatment (EAC, *P*<0.001; AC, *P*<0.001; SHAM, *P=*0.078). The upper trapezius muscle presented a significant improvement in pain intensity in all groups for right upper trapezius (EAC, *P*<0.001; AC, *P=*0.025; SHAM, *P=*0.038) and in the EAC for left upper trapezius (*P*<0.001). Post-treatment comparisons indicated significantly lower pain on both sides of the upper trapezius in the EAC group compared to SHAM (right *P=*0.030; left *P=*0.015).

### Follow-up (Figure 2D, E, F)

A comparison of the volunteers who attended the follow-up evaluation showed no pre-treatment difference between the groups (general pain: *P=*0.581, right upper trapezius: *P=*0.761, left upper trapezius: *P=*0.844). In the post-treatment evaluations, the EAC showed significantly lower pain intensity on both sides of the upper trapezius (*P*<0.05). There were no differences among groups at follow-up.

### Multiple comparisons

A generalized linear mixed model analysis for two factors (group and time of evaluation) showed a decrease in general pain in the EAC and AC groups (*P*<0.05) and a decrease in pain intensity in the right and left upper trapezius for the EAC group (*P*<0.05), but not for the AC and SHAM groups. Despite this finding, no significant intragroup difference was noted in the follow-up data (Figure 2D, E, F).

### Range of motion

There were no significant differences in basal values among the three groups for the movements. After treatment, a significant increase in rotation to the right (*P=*0.049) was observed in the EAC group, and significant increases in inclination to the right (*P=*0.005) and rotation to the right (*P=*0.032) were observed in the AC group. No changes occurred in the SHAM group (Table 1). Regarding the follow-up evaluation, only the increase in inclination to the right in the AC group was maintained (*P*>0.05; Table 2). An analysis of generalized linear mixed models failed to identify intragroup differences, either after treatment or at follow-up (*P*>0.05). Moreover, the correlations between VAS and fleximetry data were not significant (*P*>0.05). Some correlations among cervical range of motion that were not significant before treatment were significant on post-treatment evaluation: flexion vs rotation to the left (r=0.32); flexion vs rotation to the right (r=0.26); extension vs rotation to the right (r=0.30); and extension vs inclination to the right (r=0.53).

**Table 1:** Intra- and intergroup comparisons of range of motion pre-treatment and post-treatment (eight sessions; means±standard deviations).

		**EAC (n=20)**		**AC (n=21)**		**SHAM (n=19)**	
Flexion	PRE	53.95±7.78	*P=*0.218	*54.00±18.00*	*P=0.054*	52.63±11.39	*P=*0.213
	POST	56.15±9.04		*58.00±8.00*		55.68±5.64	
Extension	PRE	64.85±10.06	*P=*0.844	66.76±12.31	*P=*0.356	*70.00±14.00*	*P=0.927*
	POST	64.40±10.63		69.05±13.59		*67.00±13.00*	
Inclination to the right	PRE	43.40±6.29	*P=*0.587	41.81±6.20*	***P*** **=0.005**	41.21±7.66	*P=*0.152
	POST	44.20±6.64		44.86±5.79*		43.26±6.89	
Inclination to the left	PRE	41.65±7.11	*P=*0.220	40.67±6.92	*P=*0.060	42.79±9.67	*P=*0.751
	POST	43.95±7.78		42.95±7.58		43.37±8.01	
Rotation to the right	PRE	69.50±9.43	*P=*0.051	70.67±7.92*	***P*=0.032**	69.84±9.63	*P=*0.117
	POST	73.70±10.25		74.00±7.73*		72.84±7.94	
Rotation to the left	PRE	69.05±11.36*	***P*=0.049**	70.86±9.70	*P=*0.095	70.05±10.89	*P=*0.917
	POST	73.30±8.29*		73.19±10.18		70.26±12.43	

PRE= Pre-treatment, POST= Post-treatment; *in the same column means intragroup differences (bold fonts); Intergroup comparisons: no significant differences (*P>*0.05); Normal fonts: Paired t-test (means and standard deviations); Italic fonts: Wilcoxon's signed rank test (medians and interquartile deviations).

**Table 2:** Intra- and intergroup comparisons of range of motion in 45 volunteers who attended the follow-up evaluation (pre-treatment, post-treatment: eight sessions; follow-up: one month).

		**EAC (n=17)**		**AC (n=15)**		**SHAM (n=13)**	
Flexion	PRE	53.41±7.49	*P=*0.415	52.27±9.99	*P=*0.079	52.39±12.06	*P=*0.263
	POST	55.77±9.34		56.20±8.28		55.79±5.02	
	FU	55.06±7.66		55.60±10.75		57.15±7.67	
Extension	PRE	64.24±8.15	*P=*0.997	68.00±12.04	*P=*0.244	71.70±9.40	*P=*0.853
	POST	64.18±9.11		72.60±13.30		70.62±9.20	
	FU	64.06±9.89		70.87±12.80		70.00±7.82	
Inclination to the right	PRE	43.53±6.62	*P=*0.859	40.00±9.50^A^	P=0.002	43.20±7.71	*P=*0.797
	POST	44.00±7.12		45.00±8.00^B^		44.30±7.30	
	FU	44.35±4.99		45.00±9.50^B^		44.31±6.42	
Inclination to the left	PRE	41.82±6.61	*P=*0.166	41.60±6.05	*P=*0.068	45.00±10.10	*P=*0.981
	POST	43.53±8.29		44.87±6.91		44.46±7.54	
	FU	44.94±6.05		43.67±5.95		45.00±6.06	
Rotation to the right	PRE	*69.00±10.00*	*P=*0.079	69.53±8.37	*P=*0.100	70.84±10.39	*P=*0.081
	POST	*76.00±13.00*		73.47±7.93		75.46±9.63	
	FU	*71.00±14.00*		73.73±7.18		76.308±6.90	
Rotation to the left	PRE	68.24±10.48	*P=*0.135	69.80±10.44	*P=*0.398	71.00±12.60	*P=*0.399
	POST	72.61±6.82		72.20±10.23		71.08±3.97	
	FU	72.41±9.89		72.60±9.29		74.23±12.11	

PRE= pre-treatment; POST= post-treatment; FU=Follow-up; Different uppercase letters in the same column = intragroup differences (italic bold fonts); Intergroup comparisons: no significant differences; Normal fonts: one-way repeated measures analysis of variance (means and standard deviations); Italic fonts: Friedman's repeated measures analysis of variance (medians and interquartile deviations).

Considering the obtained data, the hypothesis was partially confirmed.

### Additional Data Form

The monitored occurrences on previous days and between sessions were similar among groups (Table 3). Compared with the EAC and AC groups, the SHAM volunteers showed an increase in medication use and headache frequency in the last week of treatment and before the post-treatment evaluation. While the EAC and AC volunteers showed a decrease in headache frequency, the SHAM volunteers reported more headaches during the later sessions compared with the first sessions. Positive and negative life experiences remained almost unchanged for all groups. Neck and shoulder pain frequency decreased slightly in all groups, with an increase in shoulder pain between the last session and the post-treatment evaluation for the AC group.

**Table 3:** Percentage of volunteers who reported medication use, positive or negative life experiences, headache or neck and shoulder pain in the post-treatment evaluation (n=60).

**Sessions**		**1 (n) %**	**2 (n) %**	**3 (n) %**	**4 (n) %**	**5 (n) %**	**6 (n) %**	**7 (n) %**	**8 (n) %**	9* (n) %
Medication use	SHAM	(9) 47	(7) 37	(5) 26	(7) 37	(2) 11	(3) 16	(2) 11	(6) 32	(9) 47
	EAC	(8) 40	(5) 25	(5) 25	(8) 40	(6) 30	(1) 5	(2) 10	(3) 15	(6) 30
	AC	(13) 62	(7) 33	(3) 14	(3) 14	(5) 24	(6) 29	(2) 10	(7) 33	(6) 29
Positive life experiences	SHAM	(2) 11	(0) 0	(2) 11	(1) 5	(0) 0	(1) 5	(2) 11	(1) 5	(2) 11
	EAC	(3) 15	(1) 5	(0) 0	(1) 5	(0) 0	(3) 15	(2) 10	(1) 5	(2) 10
	AC	(1) 5	(0) 0	(2) 10	(2) 10	(2) 10	(0) 0	(3) 14	(1) 5	(3) 14
Negative life experiences	SHAM	(8) 42	(5) 26	(3) 16	(5) 26	(3) 21	(6) 32	(3) 21	(7) 37	(6) 32
	EAC	(5) 25	(3) 15	(5) 25	(5) 25	(3) 15	(3) 15	(1) 5	(2) 10	(2) 10
	AC	(6) 29	(6) 29	(4) 19	(3) 14	(5) 24	(5) 24	(3) 14	(2) 10	(7) 33
Headache	SHAM	(8) 42	(11) 58	(7) 37	(5) 26	(6) 32	(7) 37	(8) 42	(12) 63	(15) 79
	EAC	(12) 60	(8) 40	(8) 40	(10) 50	(10) 50	(6) 30	(9) 45	(6) 30	(8) 40
	AC	(16) 76	(15) 71	(7) 33	(8) 38	(8) 38	(11) 52	(7) 33	(8) 38	(10) 48
Neck pain	SHAM	(13) 68	(11) 58	(9) 47	(9) 47	(8) 42	(10) 53	(10) 53	(10) 53	(9) 47
	EAC	(13) 65	(11) 55	(13) 65	(11) 55	(10) 50	(6) 30	(8) 40	(11) 55	(8) 40
	AC	(16) 76	(12) 57	(10) 48	(13) 62	(11) 52	(13) 62	(10) 48	(11) 52	(13) 62
Shoulder pain	SHAM	(19) 100	(16) 84	(12) 63	(13) 68	(15) 79	(11) 58	(13) 68	(14) 74	(11) 58
	EAC	(16) 80	(16) 80	(16) 80	(14) 70	(12) 60	(12) 60	(9) 45	(13) 65	(12) 60
	AC	(20) 95	(15) 71	(17) 81	(16) 76	(11) 52	(11) 52	(12) 57	(13) 62	(11) 52

* post-treatment evaluation.

## Discussion

This study aimed to identify whether EAC and AC are effective for treating pain and increasing range of motion in women with myofascial pain in the upper trapezius, with SHAM acupuncture as a control. The treatment did not intend to inactivate a specific MTrP, but to reestablish balance, stop stagnant energy, decrease pain, and therefore improve muscle function. Only women were included as they are more likely to develop neck pain than men^5^^,^ 6. Both the paired data and the results of the intergroup comparisons indicated that EAC and AC contribute more to the decrease in myofascial pain than SHAM acupuncture does. The fact that the EAC group presented significantly lower pain on both sides than SHAM after the respective treatments, whereas no difference was found between AC and SHAM, suggests that EAC treatment had an advantage with respect to pain intensity. Intergroup analysis for two factors showed that EAC improved general and local pain in the right and left upper trapezius, whereas AC was effective only for general pain, indicating better analgesic effects of EAC. In this sense, the analgesic effect of transcutaneous electrical acupoint stimulation^22^, which differs from EAC by the presence of a transcutaneous electrode instead of a needle, has already been described. An increase in blood flow after the use of transcutaneous electrical nerve stimulation within the upper trapezius muscle has also been reported^23^. Therefore, it is possible that the use of EAC may increase blood flow and remove the chemical mediators from the MTrP area, thereby facilitating a mechanical relaxation of the MTrP taut band. Although AC has showed a superior analgesic effect compared with SHAM or placebo^8^, the data in the present study suggest that the EAC group had electrical analgesic effects in addition to the needle acupoint stimulation effect of AC, therefore presenting better results. Accordingly, it was demonstrated that EAC reduced the use of opioid-like medication by chronic pain patients^11^ and also decreased pain intensity and increased the pressure pain threshold in women with myofascial pain in the upper trapezius after eight sessions of EAC applied to the same acupoints^12^. Regarding the analgesic effect of needling, although multiple needle insertion has been found to be better than simple needling insertion as in AC and EAC, myofascial trigger point irritability was found to be suppressed after remote acupuncture treatment, as considered by Chou et al.^24^. Therefore, the decrease in pain intensity in these treated groups could have been partially related to this suppression.

The treated groups also showed better results than SHAM with respect to range of motion. Although the decrease in pain would lead to a decrease in muscular stiffness and an increase in range of motion, no correlation between VAS and fleximetry data was found, corroborating a recent cohort study evaluating 4,293 subjects, which found a significant difference in pain intensity but not in cervical range of motion^25^, and agreeing with Gemmell and Hilland^26^.

Bilateral contraction of the upper trapezius muscle extends the neck and unilateral contraction flexes the neck, inclining the head to the same side and rotating it to the opposite side^16^. Thus, the improvement in myofascial pain in the upper trapezius would be expected to improve function, increasing the range of motion as described above. However, the increase in inclination to the right that was observed after treatment with AC and maintained until the follow-up evaluation can be related to the shortening of right upper trapezius fibers and the stretching of left upper trapezius fibers^27^. Although one side's function depends on the other side's function, the expected significant improvement in the inclination to the left was not obtained, reflecting the complexity of interpreting each component of neck movements separately. Moreover, it should be noted that rotation to the right almost presented significant difference for EAC and flexion for AC (Table 1). In this sense, we found three moderate positive correlations between movements related to upper trapezius in post-treatment that were absent in pre-treatment. These results might suggest integrated improvement in muscle function despite the absence of significant changes in some cervical movements.

Currently, the number of studies comparing individuals with nonspecific neck pain to the normal population is insufficient to allow conclusions about any specific physical dimensions related to nonspecific neck pain^28^, which can be caused by a mechanical or myofascial problem. Furthermore, available research comparing range of motion assessment tools shows that despite the high reproducibility and reliability of some methods, average values vary greatly depending on the instrument used^29^. Often, data findings represent healthy individuals^6^ in other age groups or include both genders^29^.

Nevertheless, paired data showed some improvement in the cervical range of motion of the two treated groups indicating the efficacy of AC and EAC compared to SHAM, which presented no significant changes. Despite this, intergroup analyses have failed to detect differences between groups. It is possible that both EAC and AC have contributed, albeit slightly, to the relaxation of the contracted fibers, however it is important that further studies include stretching exercises to investigate the benefits of the combination of both treatments.

Differences in muscle activity between the dominant and non-dominant side have been shown^30^, probably influencing the effects of treatment for muscle function. The contraction of the right upper trapezius promoted a significant increase (P<0.05) in inclination to the right (AC) and rotation to the right (EAC and AC). The left-handed volunteer (EAC) was the only one presenting improvement in rotation to the left.

The homogeneous distribution according to oral contraception could minimize the effects of hormonal fluctuations. Also, the additional data form was helpful, among other things, for monitoring factors such as the frequency of medication use and for showing that pain relief was not caused by an increased use of analgesics. There was little variation in reported positive and negative life experiences, which do not seem to have influenced the results. The percentage of headache complaints, possibly the referred pain from myofascial pain in the upper trapezius, was lower for the EAC and AC groups and higher for the SHAM group, in accordance with the data obtained.

Limitations must be addressed, such as the needle insertion 1 cm away from acupoints in the SHAM group but still in the dysfunctional region, possibly having some effect on the pain. Conversely, the effect of needle insertion could be present in all treated groups, thus weakening the respective influence. Fifteen volunteers did not attend the follow-up session, however this fact is inherent to clinical research. On the other hand, the initial sample attended all sessions accordingly, supporting the findings.

## Conclusion

Both AC and EAC were superiorly effective in reducing myofascial pain compared with SHAM acupuncture. Nonetheless, EAC was better than AC for pain relief in the studied sample. There are indications that those treatments can assist in increasing range of motion, albeit subtly.
